# Endangered plant growth was influenced by soil microbial life-history strategies across elevations

**DOI:** 10.3389/fmicb.2026.1922651

**Published:** 2026-10-15

**Authors:** Qiumei Teng, Ying Wang, Shihong Lyu, Jinxing Wei, Guangping Xu, Tao Ding

**Affiliations:** 1 Guangxi Key Laboratory of Plant Conservation and Restoration Ecology in Karst Terrain, Guangxi Institute of Botany, Guangxi Zhuang Autonomous Region and Chinese Academy of Sciences, Guilin, China; 2 Guangxi Guilin Urban Station (City Type) of Ecological Quality Comprehensive Monitoring Station of Ministry of Ecology and Environment, Guilin, China; 3 Guangxi Lijiangyuan Forest Ecosystem Research Station, Guilin, China

**Keywords:** *Abies yuanbaoshanensis*, elevation, endangered plant, microbial life strategy, soil microbial community

## Abstract

Elevations can influence belowground microbial diversity, network complexity, life history strategy, and the growth of endangered plants. However, how elevation affects endangered plant growth by modulating soil microbial network complexity and life-history strategies remains poorly understood. This study focused on *Abies yuanbaoshanensis* and systematically examined tree height, diameter at breast height (DBH), soil physicochemical properties, enzyme activity, and microbial communities at different elevations (1,750 m, 1,850 m, 1,950 m, and 2,050 m). The results showed that tree height and DBH were highest at 1,750 m and lowest at 1,850 m compared to other elevations. At an elevation of 1,850 m, soil total phosphorus (TP), total potassium content, and fungal and bacterial diversity were the lowest, while soil water content and fungal oligotrophs/copiotrophs ratios were the highest. The complexity of bacterial-fungal networks increased with elevation. Structural equation modeling suggests a potential chained correlative pathway, wherein elevation was associated with shifts in soil water content, which in turn correlated with alterations in TP content, subsequently showing associative relationships with the abundance of oligotrophic fungi and microbial network complexity, and finally exhibiting links to changes in plant growth. These findings underscore that soil physicochemical properties and microbial communities jointly determine the growth of endangered mountain plants along altitudinal gradients. Although bacterial and fungal diversity exert distinct effects on the growth of endangered plants, their life history strategies should be considered a critical factor in the management of these species. This research provides a new perspective for the *ex situ* conservation and *in situ* management of endangered plants.

## 1 Introduction

*Abies yuanbaoshanensis* is a China-endemic endangered fir listed as a National First-Class Key Protected Wild Plant, surviving only at elevations of 1,750–2,050 m on Mount Yuanbao in northern Guangxi (Ren et al., 2012; Tang et al., 2024). As a Quaternary glaciation relict, it holds significant value for studying floral evolution and past climate in southern China (Tang et al., 2024). Ongoing global warming has intensified climate anomalies in its high-elevation habitat, leading to continuous shrinkage and fragmentation of suitable habitats; fewer than 900 wild individuals remain at present (Tang et al., 2024). Elevation drives systematic changes in soil properties by modulating temperature, precipitation, and light, thereby affecting soil microbes and plant growth (Midolo et al., 2019; Wang et al., 2025). Rhizosphere microbes are key to host nutrient acquisition, stress tolerance, and growth regulation (Philippot et al., 2013; Liu B. et al., 2025). However, how elevation modulates the interplay between soil physicochemical properties and microbial communities to influence the growth of *A. yuanbaoshanensis* remains unclear, a knowledge gap that severely impedes targeted conservation efforts.

In contrast to harsh high-elevation environments, low-elevation areas typically offer a warmer climate, higher CO_2_ partial pressure, and more fertile soils, which together promote vigorous plant growth (Midolo et al., 2019; Kiełtyk, 2024). Accordingly, decreasing elevation generally enhances tree height, internode length, leaf area, and biomass (Iakovoglou et al., 2019; Cruz-Maldonado et al., 2021). However, this pattern is not universal. Wang et al. (2025) found that traits such as tree height and diameter at breast height peaked at mid-elevations. Such trait variation influences survival, competition, reproduction, and population dynamics. Thus, investigating trait variation along elevational gradients is crucial for understanding plant adaptation to stressful environments and predicting species range shifts under climate change.

Soil carbon, nitrogen, and phosphorus contents and their stoichiometric ratios are key indicators of soil fertility, directly influencing plant growth (Li et al., 2014). Elevation shapes the spatial distribution of soil properties by regulating hydrothermal conditions, weathering intensity, and erosion-deposition processes (Luo et al., 2024). Low-elevation areas typically exhibit higher phosphorus availability due to enhanced weathering of phosphorus-containing minerals under warmer and more humid conditions and to the accumulation of eroded upslope materials (Güzel et al., 2025). Adequate phosphorus supports root development, photosynthesis, and biomass accumulation, promoting greater tree height and leaf area, whereas phosphorus deficiency restricts growth (Poudyal et al., 2021). The soil C:N:P ratio, which varies with elevation, reflects nutrient limitation and reflects microbial activity and community assembly (Feng et al., 2021). However, how soil stoichiometric ratios affect the growth of endangered plants along elevational gradients in mountain ecosystems remains poorly understood.

Soil microbial community plays a key role in plant growth (Siles and Margesin, 2016). Microbial alpha diversity, community assembly, and co-occurrence network complexity vary with elevation due to shifts in microbial taxa, soil properties, and climatic conditions (Zhang J. et al., 2025; Liu B. et al., 2025; Zhou et al., 2026). However, findings are inconsistent: some studies report stochastic dominance in bacterial assembly at low elevations, while others find that stochastic processes prevail along the entire elevational gradient (Li et al., 2024; Zhang Z. H. et al., 2025). Network complexity often declines at higher elevations, though network stability may peak at mid-elevations (Ma et al., 2025; Zhang J. et al., 2025). These elevational patterns are driven by trait trade-offs in resource use, growth yield, and stress tolerance (Piton et al., 2023). Trait-based life history strategies, particularly the r/K selection framework, are critical to soil structure and plant productivity (Wang et al., 2022). Generally, r-strategists (copiotrophs) dominate nutrient-rich or disturbed environments, while K-strategists (oligotrophs) prevail in stable, resource-poor conditions (Fierer et al., 2007). Linking microbial life strategies to plant growth can help reveal soil community structure and function relationships (Zhou et al., 2026). Nevertheless, how elevation affects microbial life history strategies and subsequently influences the growth of endangered plants remains unclear.

This study focuses on *A. yuanbaoshanensis* to systematically analyze plant traits, soil physicochemical properties, and microbial community characteristics along an elevational gradient. Our objectives were to explore the responses of *A. yuanbaoshanensis* to biotic and abiotic factors at varying elevations, identify its optimal elevational range for growth, and reveal the mechanisms underlying its endangerment. We hypothesize that (1) *A. yuanbaoshanensis* would exhibit a non-linear response along the elevational gradient; (2) elevation differentially affects soil physicochemical properties and microbial life history strategies; and (3) interactions between soil physicochemical properties and microbial life history strategies influence the growth of *A. yuanbaoshanensis*. The findings will provide a theoretical basis for the *in situ* conservation and *ex situ* introduction of *A. yuanbaoshanensis*, and will enhance our understanding of plant-soil-microbe interactions in mountain forest ecosystems.

## 2 Materials and method

### 2.1 Study sites

The study area was located in the Yuanbaoshan National Nature Reserve in Rongshui Miao Autonomous County, Liuzhou City, Guangxi province, China (109°7′-109°13′E, 25°2′-25°32′N; Figure 1). The region has a subtropical monsoon climate, characterized by abundant heat and precipitation: the annual average temperature is 16.4 °C, accompanied by 2,379 mm of rainfall and a frost-free period of 288 days. Rainfall is mainly concentrated between April and August, making this the region with the highest rainfall in Guangxi. Yuanbaoshan is often shrouded in mist and has relatively high humidity, especially in high elevations areas where the average humidity exceeds 80%. In winter, snow and ice are common. *A. yuanbaoshanensis* is found only around Yuanbaoshan at elevations of 1,750–2,050 m. The main associated tree species include *Tsuga chinensis, Taxus wallichiana var*. mairei, *Cephalotaxus hainanensis, Fagus lucida, Lithocarpus cleistocarpus*, and *Pterostyrax psilophyllus*. Bamboo is widespread under the forest canopy, and the area is densely covered with lichens, mosses, and other understory plants. The soil is classified as mountain yellow soil according to the FAO/UNESCO soil classification system.

**Figure 1:**
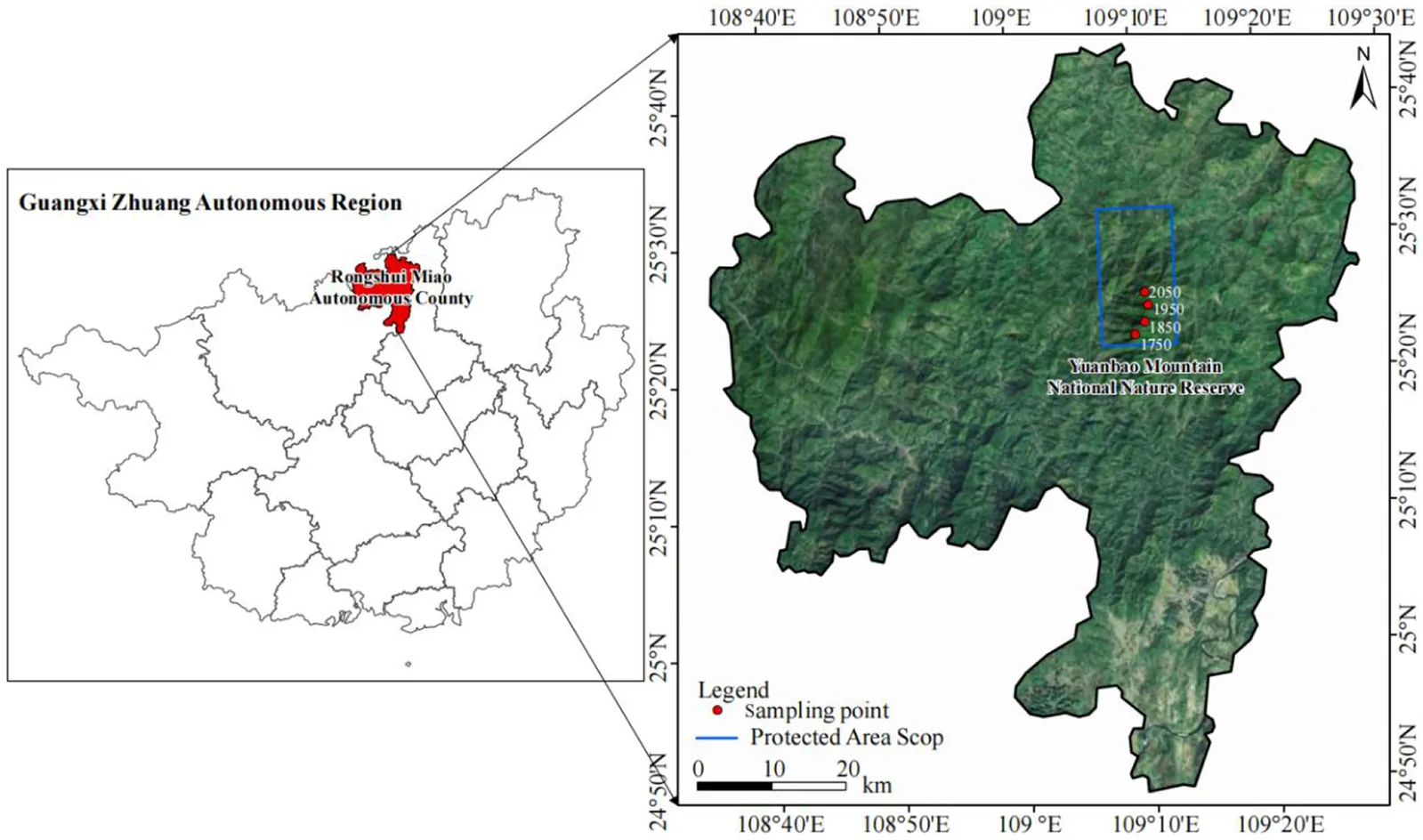
Location of sampling sites.

### 2.2 Sample collection

Sampling was conducted in December 2022. Based on the growth and distribution of *A. yuanbaoshanensis*, four elevations were selected: 1,750, 1,850, 1,950, and 2,050 m. Due to the extremely small population size of this rare and endangered species in the wild, combined with natural constraints and species-specific characteristics, sampling may be restricted to a limited number of available individuals. This prevented us from meeting conventional sampling standards, and consequently may have contributed to the observed variation in growth traits among the sampled trees (Hu P. et al., 2023). At each elevation, three 50 m × 50 m plots were established, and three vigorous, similar *A. yuanbaoshanensis* individuals were selected from each plot as standard trees. The tree height and DBH of each standard tree were measured. Plant height and DBH measurements from the three trees within each plot were averaged within each plot prior to statistical analysis, resulting in a total of 12 plant samples (4 elevations × 3 plots), with *n* = 3 per elevation. Soil samples were then collected from the east, south, west, and north directions around each standard tree. After removing the surface litter and humus layer at approximately 0.5 m from the trunk, soil samples were obtained using a soil auger. The samples from the three standard trees within a plot were mixed into one composite sample, resulting in a total of 12 soil samples (4 elevations × 3 plots), with *n* = 3 per elevation. Fresh soil samples were brought back to the laboratory and then cleared of visible roots and other residues. The samples were then divided into three parts: one part was immediately used to measure soil enzyme activity, another part was stored in a −80 °C freezer for DNA extraction, and the third part was air-dried for analysis of soil physicochemical properties.

### 2.3 Soil physicochemical properties and enzyme activities

Soil pH was measured using a pH meter (FE20K; Mettler Toledo, Greifensee, Switzerland). Soil water content (SWC) was determined by oven-drying at 105 °C. Soil organic carbon (SOC) was quantified using wet oxidation with the dichromate colorimetric method. Total nitrogen (TN) was analyzed using an Elementar Vario MAX Cube (Elementar, Hanau, Germany). Total phosphorus (TP) and total potassium (TK) were measured by the ammonium molybdate method and a flame spectrophotometer method, respectively.

Urease activity was assayed using the indophenol blue colorimetric method (Teng et al., 2023). Briefly, 5 g of fresh soil was incubated with 10 mL of 10% urea solution and 20 mL of citrate buffer (pH 6.7) at 37 °C for 24 h. After incubation, the filtrate was reacted with sodium phenolate and sodium hypochlorite, and the absorbance was measured at 578 nm. Urease activity was expressed as U ml^−1^ (1 U = 1 μg ammonium nitrogen released per ml per hour). Invertase activity was determined using the 3,5-dinitrosalicylic acid (DNS) colorimetric method (Teng et al., 2023). Fresh soil (5 g) was incubated with 15 mL of 8% sucrose solution and 5 mL of phosphate buffer (pH 5.5) at 37 °C for 24 h. The reducing sugars produced were quantified by reaction with DNS reagent, with absorbance measured at 508 nm. Invertase activity was expressed as U ml^−1^ (1 U = 1 μg glucose released per ml per hour). Phosphatase activity was quantified using the disodium phenyl phosphate colorimetric method (Tabatabai and Bremner, 1969). Fresh soil (5 g) was incubated with 20 mL of disodium phenyl phosphate solution and 5 mL of borate buffer (pH 10.0) at 37 °C for 24 h. The released phenol was determined spectrophotometrically at 510 nm. Phosphatase activity was expressed as U ml^−1^ (1 U = 1 μg phenol released per ml per hour). Dehydrogenase activity was measured using the triphenyltetrazolium chloride (TTC) colorimetric method (Casida et al., 1964). Fresh soil (5 g) was incubated with 5 mL of 1% TTC solution and 2 mL of Tris-HCl buffer (pH 7.6) at 37 °C for 24 h in the dark. The triphenylformazan (TPF) produced was extracted with methanol and quantified spectrophotometrically at 485 nm. Dehydrogenase activity was expressed as U ml^−1^ (1 U = 1 μg TPF released per ml per hour).

### 2.4 Soil DNA extraction and high-throughput sequencing

Total DNA was extracted from 0.5 g of soil using the ALFA Soil DNA Extraction Kit (FINDROP, Guangzhou, China), following the manufacturer's instructions. The fungal internal transcribed spacer (ITS) region was amplified using primers ITS3-F 5′-GCATCGATGAAGAACGCAGC3′) and ITS4-R 5′-TCCTCCGCTTATTGATATGC3′). The bacterial 16S rRNA gene was amplified using primers 338F 5′-ACTCCTACGGGAGGCAGCA3′) and 806R 5′-GGACTACHVGGGTWTCTAAT3′). The PCR protocol included initial denaturation at 94 °C for 5 min, 31 cycles of denaturation (94 °C, 30 s), annealing (53 °C, 30 s), and extension (72 °C, 30 s), and a final extension at 72 °C for 8 min before holding at 12 °C. After amplification, the PCR products were purified and mixed in equal amounts. The products were then visualized by electrophoresis on a 2% agarose gel and recovered using the Axygen Gel Recovery Kit (AXYGEN, United States). Following verification on a 2% agarose gel, the samples were sent to Guangdong Meige Technology Co., Ltd (Shenzhen, China), where high-throughput sequencing was performed on the Illumina NovaSeq PE250 platform (Illumina Inc., CA, United states).

Raw sequencing data were processed using QIIME2 (v 2020.11.0). Quality filtering was performed to remove low-quality reads, and chimeric sequences were identified and removed using the vsearch plugin. High-quality sequences were then clustered into operational taxonomic units (OTUs) at a 97% similarity threshold using the q2-vsearch plugin. Representative sequences for each OTU were taxonomically assigned using the Naïve Bayes classifier trained on the SILVA database (v138) for bacterial 16S rRNA and the UNITE database (v8.0) for fungal ITS sequences, with a confidence threshold of 0.8. To account for uneven sequencing depth across samples, the OTU table was rarefied to the minimum sequencing depth (bacteria: 1,01,873 reads/sample; fungi: 79,317 reads/sample) prior to downstream analyses. All subsequent bioinformatics analyses were based on the rarefied OTU table. Raw sequencing data have been submitted to the NCBI Read Archive database and can be retrieved via accession number PRJNA1418547.

### 2.5 Community assembly and network analysis

To evaluate how elevation influences microbial community assembly patterns, we quantified the relative contributions of deterministic and stochastic processes using the phylogenetic normalized stochasticity ratio (NST). Based on the null model framework of Stegen et al. (2013) and Ning et al. (2020), NST measures average stochasticity within a group of samples using phylogenetic beta diversity. A 50% threshold was used distinguishes deterministic (NST < 50%) from stochastic (NST > 50%) assembly processes.

Co-occurrence networks were constructed separately for each elevation to provide a preliminary and descriptive visualization of potential association patterns among microbial taxa. Bacterial-fungal interaction networks were built with the MENA pipeline (http://ieg4.rccc.ou.edu/MENA/) using Random Matrix Theory (RMT). Symbiotic network construction and topological analysis were performed following Wang et al. (2018). OTUs found in less than 40% of samples were removed, thereby minimizing spurious associations. Network visualization was subsequently carried out using Gephi (version 0.9.2). To comprehensively characterize network architecture, we quantified multiple topological features, including network scale (e.g., node count, total edge count, and average degree) and structural composition (e.g., the proportion of negative links). Among these, we selected the proportion of negative links as a focal structural metric for hypothesis testing. This specific metric was prioritized not as a comprehensive proxy for overall complexity, but because it directly captures the intensity of negative associations (e.g., competition, niche differentiation, or predation resistance) that are central to our ecological questions.

### 2.6 Assessment of microbial life strategies

Bacterial life strategies were assigned at the phylum level following Leff et al. (2015) and Dai et al. (2018). Specifically, Acidobacteria, Planctomycetes, Verrucomicrobia, and Chloroflexi were considered oligotrophic taxa, whereas Actinobacteria, Gemmatimonadetes, and Bacteroidetes were categorized as copiotrophic. The oligotroph-to-copiotroph ratio (oligo/copio ratio) for bacteria was calculated as the sum of relative abundances of oligotrophic phyla divided by that of copiotrophic phyla. For fungi, Basidiomycota and Ascomycota were taken as representative of oligotrophic and copiotrophic lifestyles, respectively (Hu R. et al., 2023). Accordingly, the fungal oligo/copio ratio was defined as the relative abundance of Basidiomycota divided by that of Ascomycota (Hu R. et al., 2023).

### 2.7 Data analysis

Differences in plant traits, soil physicochemical properties, enzyme activities, and microbial traits across elevations were assessed using one-way analysis of variance (ANOVA), followed by Tukey's test at *p* < 0.05 using SPSS 22.0 (IBM, Armonk, NY, United States). Prior to analysis, the normality of the data was assessed using the Shapiro–Wilk test and the homogeneity of variance using Levene's test. All variables satisfied these assumptions (*p* > 0.05). Relationships among these variables were examined using Pearson correlation analysis in Origin 2021. Microbial alpha diversity indices (ACE, Chao1, Shannon, and Simpson) were calculated using the “vegan” package. Community compositional variation (beta diversity) was evaluated based on Bray–Curtis's dissimilarity and visualized using non-metric multidimensional scaling (NMDS). Differences in microbial communities among elevations were further tested using analysis of similarities (Anosim, *p* < 0.01). Redundancy analysis (RDA) was conducted in CANOCO 5 to explore the relationships between microbial composition and plant traits, as well as soil physicochemical variables. Based on the results of correlation analysis and RDA, structural equation modeling (SEM) was performed using AMOS 20.0 (IBM SPSS AMOS) to quantify the direct and indirect effects of soil physicochemical and microbial properties on plant growth. Model performance was evaluated using multiple fit indices, including a non-significant chi-square test (*p* > 0.05), a chi-square to degrees of freedom ratio (0 ≤ χ^2^/df ≤ 2), root mean square error of approximation (0 ≤ RMSEA ≤ 0.05), and comparative fit index (0.97 ≤ CFI ≤ 1).

## 3 Results

### 3.1 Effects of elevation on plant traits

Contrary to a simple linear expectation, plant height and DBH exhibited a pronounced non-linear trajectory along the elevational gradient. The DBH and tree height both decreased first and then increased with the increase of elevation, showing a pattern of 1,750 > 2,050 >1,950 > 1,850 m (*p* < 0.05; Figures 2a, b). The DBH at elevations of 1,750, 2,050 and 1,950 were 72.0%, 38.5%, and 56.3% higher than those at 1,850 m respectively, and the tree heights were 54.5%, 27.3%, and 47.7% higher respectively.

**Figure 2:**
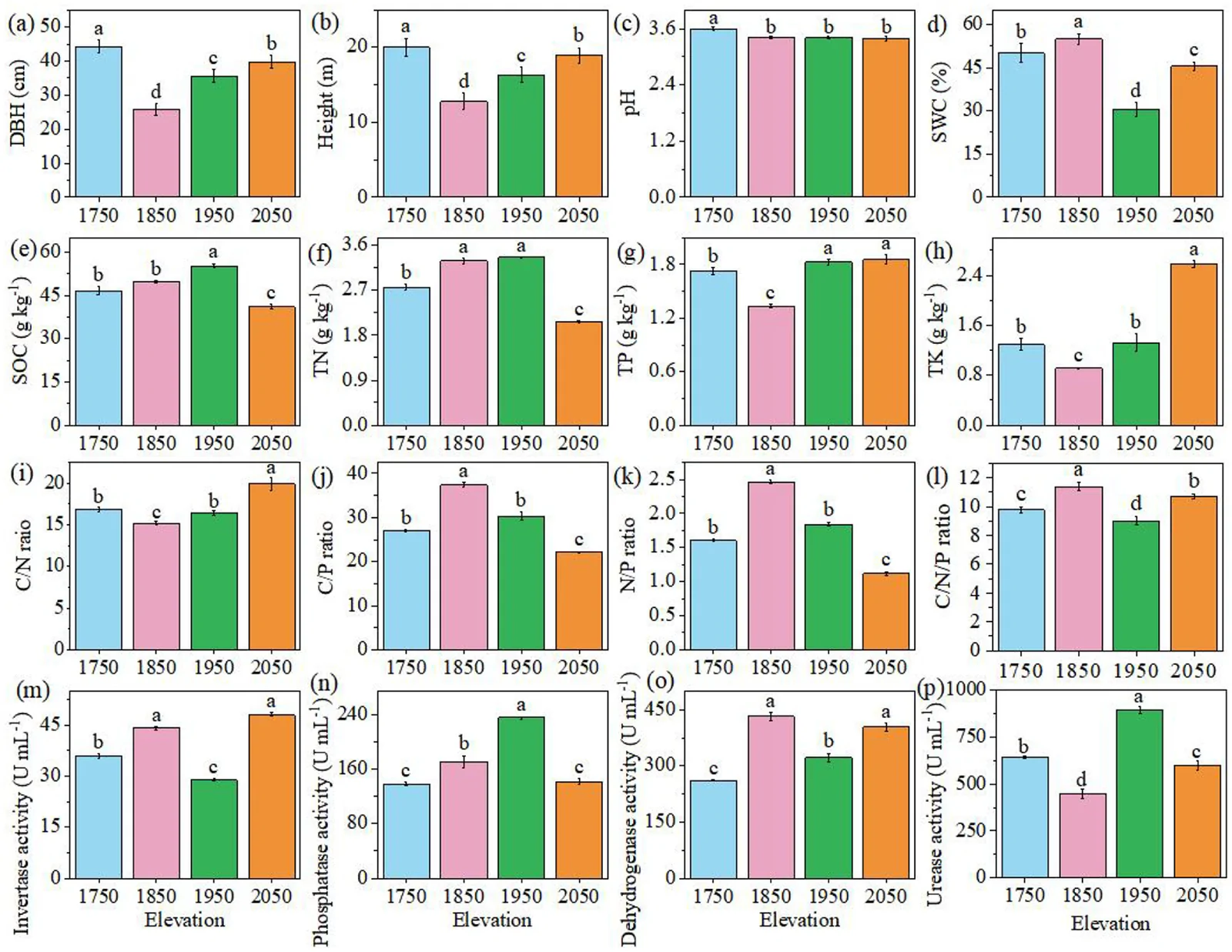
Plant traits, soil physicochemical, and enzyme activities at different elevation. **(a)** Diameter at breast height; **(b)** tree height; **(c)** soil pH; **(d)** soil water content; **(e)** soil organic carbon; **(f)** total nitrogen; **(g)** total phosphorus; **(h)** total potassium; **(i)** the ratio of SOC to TN; **(j)** the ratio of SOC to TP; **(k)** the ratio of TN to TP; **(l)** the ratio of SOC to TN to TP; **(m)** invertase activity; **(n)** phosphatase activity; **(o)** dehydrogenase activity; **(p)** urease activity. Different lowercase letters indicates significant differences among elevations (*p* < 0.05).

### 3.2 Effects of elevation on soil physicochemical properties and enzyme activities

Soil pH exhibited a decreasing trend with increasing elevation, whereas TP, TK, and the C/N ratio reached their lowest values at 1,850 m (Figures 2c, g). In contrast, C/P and N/P ratios were highest at this elevation compared with other elevations. SOC content was higher at 1950 m than other elevations (Figure 2e). TK content and the C/N ratio decreased initially and then increased with elevation, while C/P and N/P ratios showed the opposite pattern, increasing first and then declining (Figures 2h–k). Enzyme activities varied with elevation, invertase, phosphatase, and dehydrogenase activities were higher at 1,850 m than at 1,750 m, whereas urease activity was lower at 1,850 m compared with 1,750 m (Figures 2m–p).

### 3.3 Effects of elevation on soil microorganisms

Elevation has a significant impact on the composition of soil bacterial and fungal composition (Anosim: bacterial and fungal composition were respectively: *R* = 0.37, *p* < 0.01 and *R* = 0.34, *p* < 0.01; Figures 3a, b). The Chao1, Ace, and Shannon indices for both soil bacterial and fungal communities were the lowest at an elevation of 1,850 m (Figures 3c, d). At an elevation of 1,750 m, the NST of fungal was below 0.5, whereas at all other elevations the NST of both fungal and bacterial communities exceeded 0.5 (Figures 3e, f).

**Figure 3:**
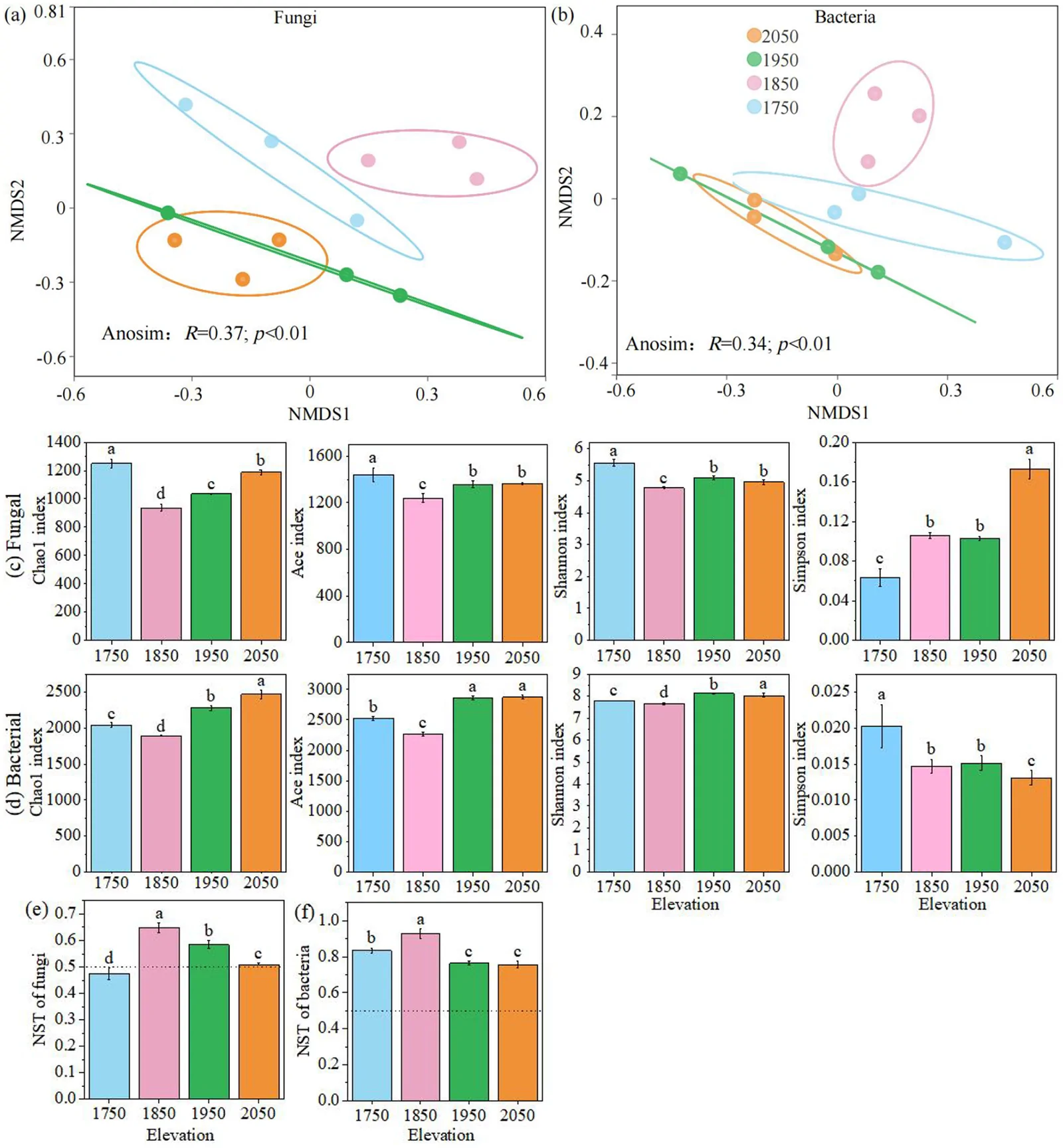
Soil microbial diversity **(a–d)** and communities' assembly processes **(e, f)**. Different lowercase letters indicate significant differences among elevations (*p* < 0.05).

Ascomycota and Basidiomycota accounted for 80% of the total soil fungal abundance (Figure 4a), while Acidobacteria, Proteobacteria, and Actinobacteria comprised 80% of the total soil bacterial abundance (Figure 4b). The relative abundance of Basidiomycota and Actinobacteria exhibited a unimodal pattern, initially increasing and then decreasing with elevation, peaking at 1,850 m. At this elevation, the relative abundance of total copiotrophic bacteria taxa and fungal oligotrophs/copiotrophs ratio were higher, whereas the bacterial oligotrophs/copiotrophs ratio was lower compared to other elevations (Figures 4d–f). The number of nodes and edges, as well as the positive correlation ratio and average degree in bacterial-fungal networks were higher at 1,750 m compared to other elevations, while the negative correlation ratio was lower (Figure 5). The negative correlation ratio exhibited a trend of increasing with elevation.

**Figure 4:**
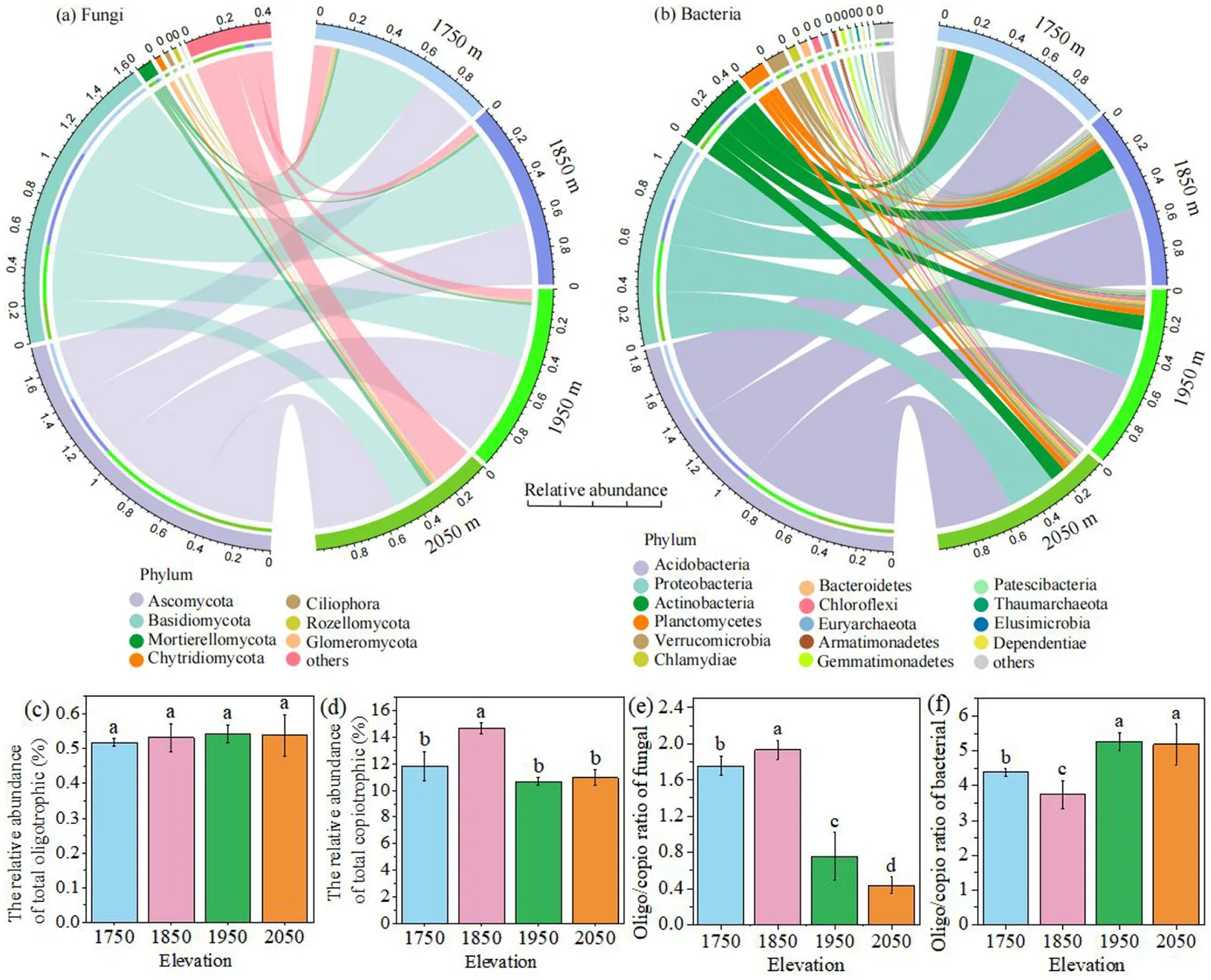
Relative abundance of bacterial and fungal communities at the phylum level **(a, b)** and life strategies **(c–f)**. Different lowercase letters indicate significant differences among elevations (*p* < 0.05).

**Figure 5:**
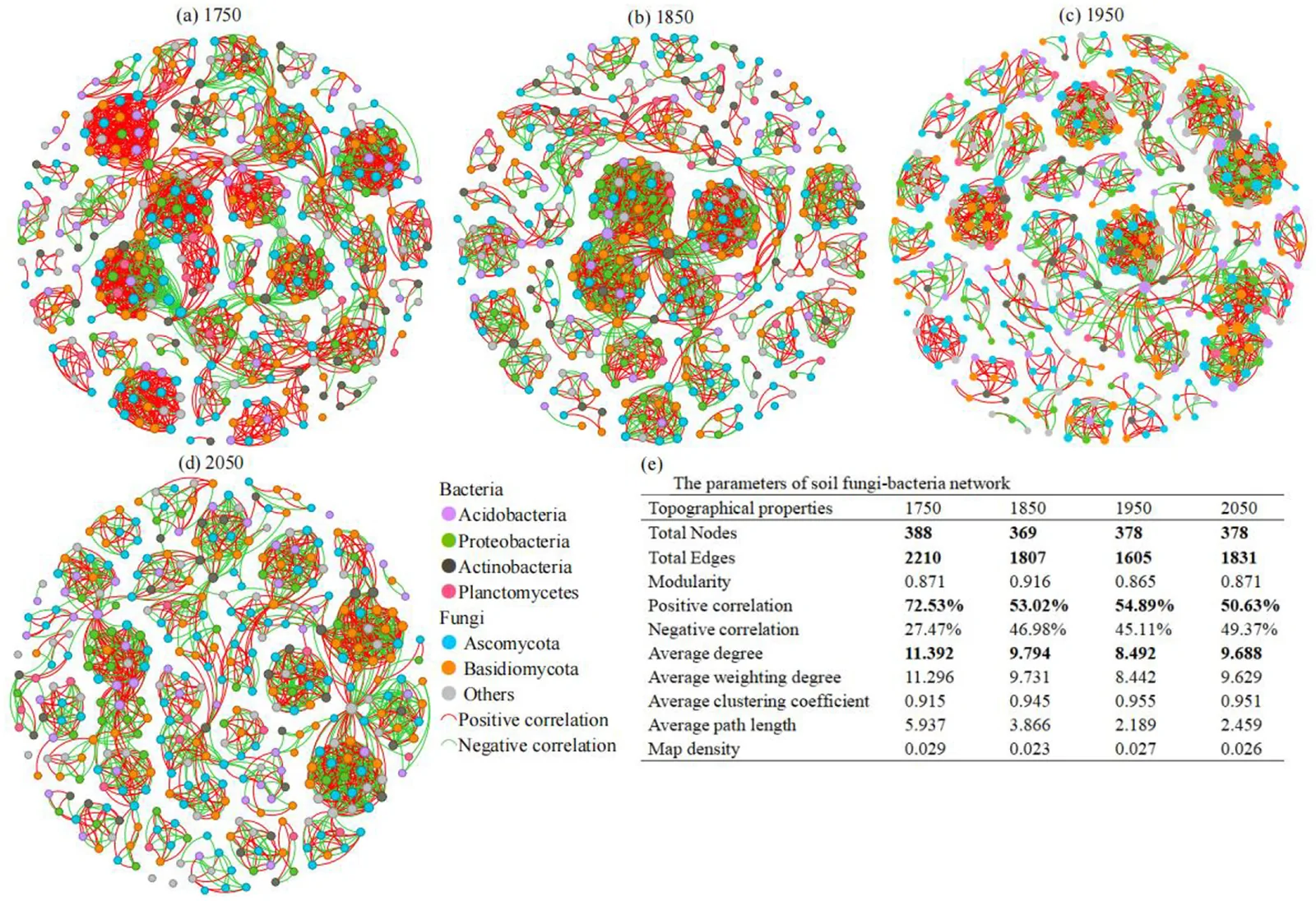
Bacterial-fungal co-occurrence networks across different elevations. Each node represents a microbial OTU, with node size proportional to its relative abundance and node color indicating taxonomic affiliation. Edges represent significant correlations, with red edges indicating positive correlations and green edges indicating negative correlations. Networks are shown for **(a)** 1,750 m, **(b)** 1,850 m, **(c)** 1,950 m, **(d)** 2,050 m, and **(e)** topological properties of networks.

### 3.4 Relationship between plant traits and soil characteristics

Pearson correlation analysis indicated that both DBH and tree height were positively associated with TK, C/N, bacterial diversity, and bacterial life strategy, and negatively associated with C/P and fungal life strategy (Figure 6a). RDA showed that both DBH and tree height have significant correlation with fungal and bacterial composition at the phylum level (Figures 6b, c). SEM revealed that TP had significant positive direct effects on bacterial diversity (β = 0.36, *p* < 0.05) and plant growth (β = 0.61, *p* < 0.05), but had a significant negative direct effect on microbial composition (β = −0.49, *p* < 0.05), fungal life history strategy (β = −0.41, *p* < 0.05), and microbial network complexity (β = −0.37, *p* < 0.05; Figure 7a). Furthermore, fungal life history strategy had the most and negative direct effects on plant growth (β = −0.52, *p* < 0.05; Figure 7a).

**Figure 6:**
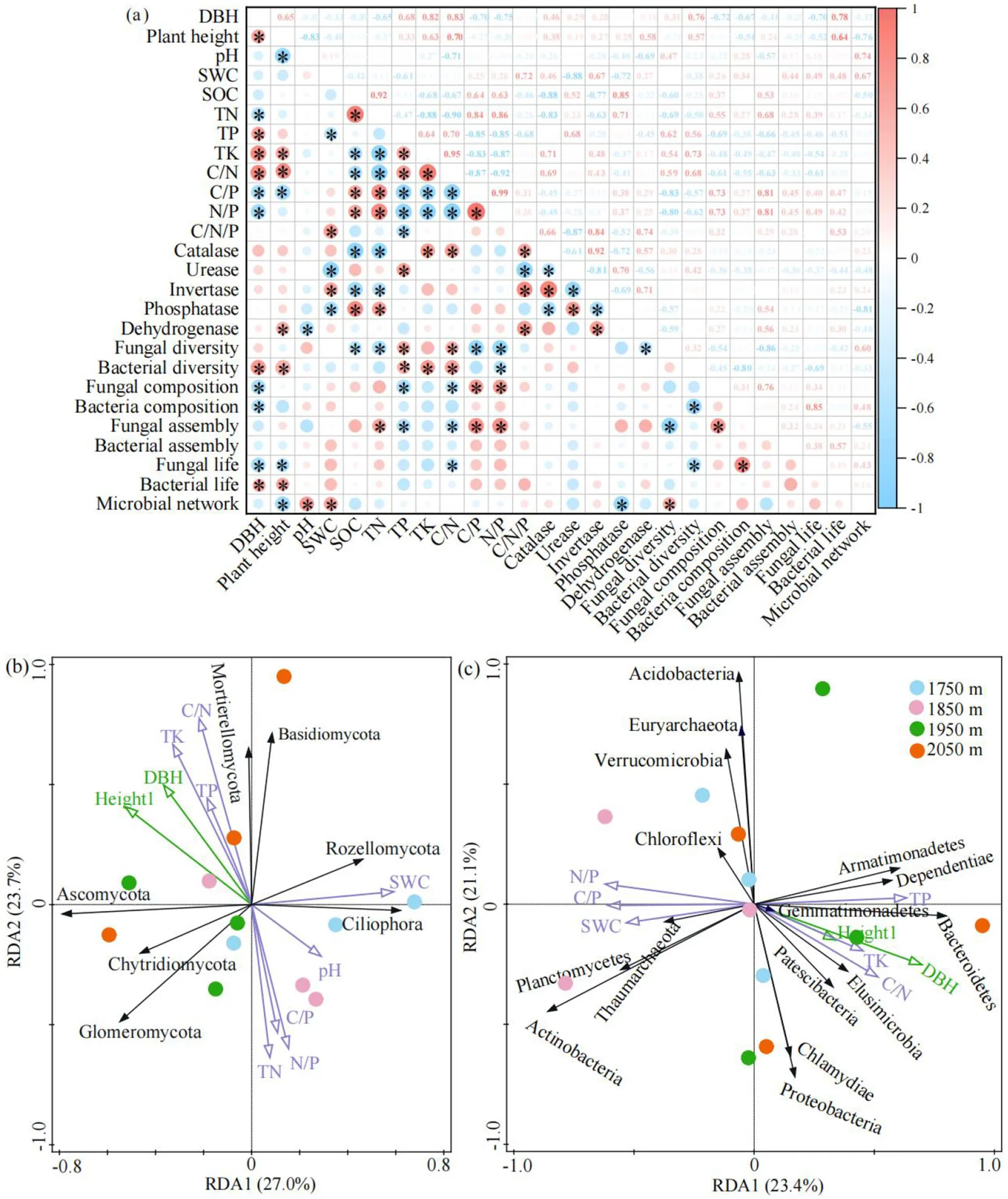
Relationship between soil characteristics and plant traits. **(a)** Pearson correlation analysis. **(b, c)** Redundancy analysis (RDA) of soil fungal and bacterial phylum richness and phenotypic traits. DBH, diameter at breast height; SWC, soil water content; SOC, soil organic carbon; TN, total nitrogen; TP, total phosphorus; TK, total potassium; C/N, the ratio of SOC to TN; C/P, the ratio of SOC to TP; N/P, the ratio of TN to TP. C/N/P, the ratio of SOC to TN to TP.

**Figure 7:**
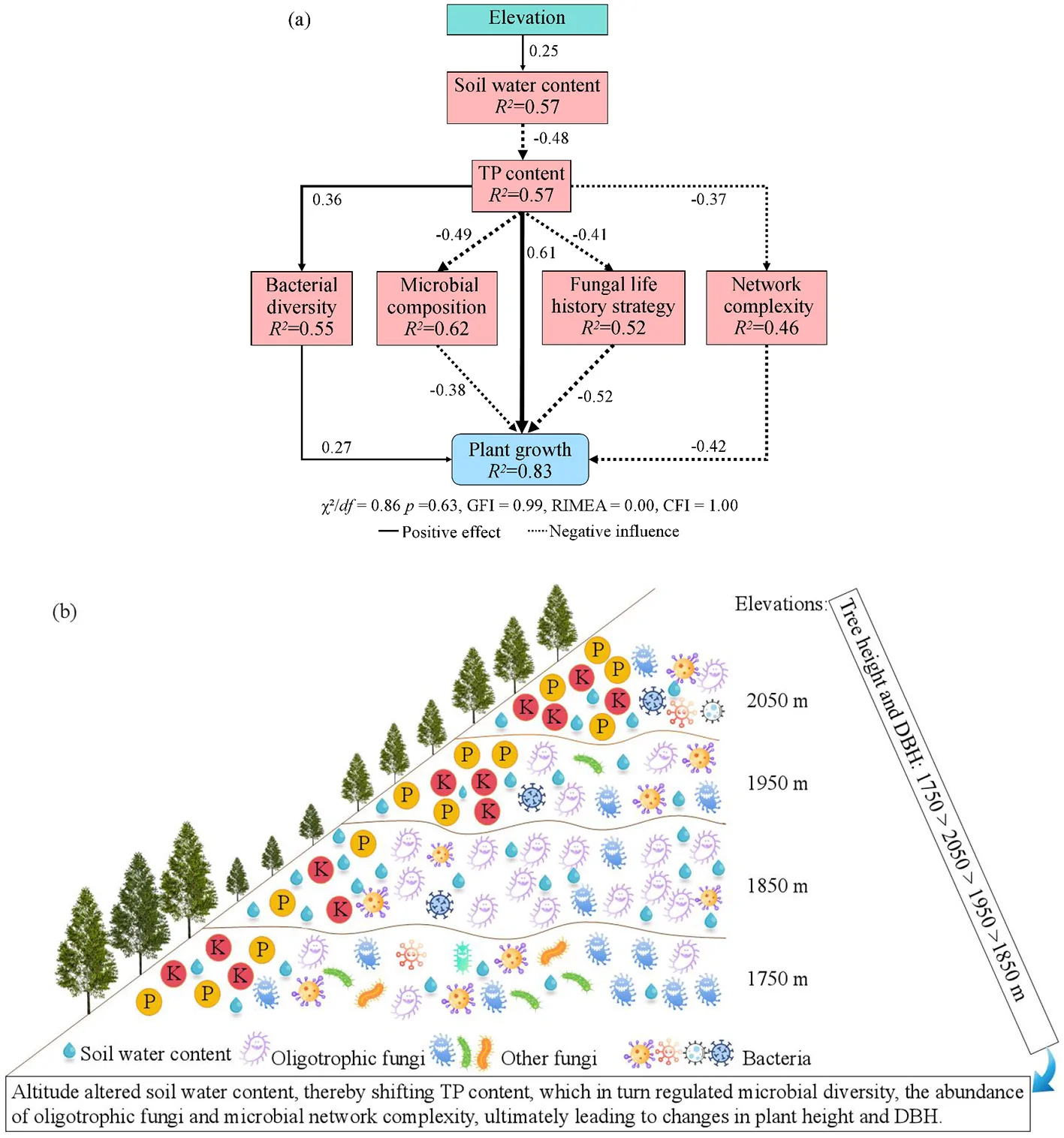
**(a)** Structural equation modeling (SEM) was used to examine how elevation, soil properties and microbial traits influence the growth of plant. **(b)** Plant traits and soil characteristics of *Abies yuanbaoshanensis* at different elevations. Bacterial diversity, chao1 index; microbial composition, including NMDS1 of fungal and bacterial; fungal life-history strategy, the oligotroph-to-copiotroph ratio (oligo/copio ratio) of fungal; network complexity, the proportion of negative links; plant growth, including diameter at breast height and plant height.

## 4 Discussion

### 4.1 Effects of elevation on plant growth

Plant growth was influenced by elevation, soil properties, and microbial communities (Kramer et al., 2000). In this study, both DBH and tree height peaked at 1,750 m, reached their minimum at 1,850 m, and then partially recovered with increasing elevation, showing an initial decline followed by a subsequent increase (Figures 2a, b). At lower elevation (1,750 m), warmer temperatures and favorable soil moisture and nutrients promoted growth (Cao et al., 2019). In the mid-elevation band (1,850–1,950 m), lower temperatures, stronger winds, and poorer soil conditions increased abiotic stress, thereby reducing growth (Popa et al., 2024). At the highest elevation (2,050 m), despite colder conditions, reduced human disturbance, lower competition, improved light, or adaptive strategies (e.g., enhanced nutrient use efficiency) may have facilitated partial growth recovery (Liu et al., 2019). Low genetic diversity and limited natural regeneration of *A. yuanbaoshanensis* may contribute to these non-linear responses along the altitudinal gradient (Farjon et al., 2011). Similarly, non-monotonic growth patterns with altitude have been observed in *Picea abies* and *Pinus canariensis* (Weigel et al., 2018; Yang et al., 2019), supporting the view that tree growth responses to elevation are generally context-dependent and region-specific rather than simply increasing or decreasing with elevation. This result only partially supports Hypothesis 1, revealing a more complex, non-linear elevational response rather than a simple low-elevation advantage.

### 4.2 Effects of elevation on soil physicochemical properties

In forest ecosystems, elevation shaped soil properties by altering temperature and moisture (Wang et al., 2025; Liu X. T. et al., 2025). Here, the 1,850 m elevation had the highest SWC, C/P, N/P, and C/N/P ratios, but the lowest TP, TK content, and C/N ratio (Figures 2d, g–l). At 1,750 m, higher temperatures enhanced evapotranspiration and thereby increased soil water loss. At 1,950 m and 2,050 m, although cooler temperatures reduced evaporation rates, soils were often shallower and rockier, leading to lower water retention (Hlaváčiková et al., 2016). The peak soil water content at 1,850 m may coincide with the regional ‘cloud belt' or ‘fog zone' described in published literature, where persistent low clouds and frequent fog deposition are reported to supply substantial non-rainfall water directly to the soil surface (Räsänen et al., 2018). This fog drip is speculated to contribute significantly to soil moisture, particularly during dry periods or when rainfall is intermittent (Räsänen et al., 2018). Moreover, mid-elevations often develop deeper, finer-textured soils with higher organic matter, enhancing water holding capacity (Liu X. T. et al., 2025).

Changes in soil moisture can alter the content of nutrient elements in the soil (Högberg et al., 2017). High soil moisture can lead to poor soil aeration, facilitating pathogen breeding, and may also cause excessive loss of certain nutrients such as P and K (Pawar et al., 2025). Therefore, one possible underlying mechanism for the low TP and TK content at 1,850 m could be the high SWC observed at this elevation. This hypothesis was supported by the negative correlation between SWC and TP (Figure 6a). This finding aligned with previous observations that water availability regulates P cycling by affecting weathering, leaching, and microbial mineralization (Zhu et al., 2026). Notably, C/P and N/P ratios at 1,850 m exceeded global forest averages, suggesting potential P limitation based on stoichiometric inference (Güsewell, 2004; Müller et al., 2017), consistent with low TP and relatively high TN at this elevation (Figures 2f, g). This N-rich, P-poor pattern reflected differential altitudinal responses: P derived from rock weathering was prone to leaching under high precipitation, whereas N was replenished via atmospheric deposition and biological fixation (Vitousek et al., 2010). Overall, our findings indicated that increasing elevation was associated with higher soil moisture content, which in turn was linked to decreased TP and TK contents.

### 4.3 Effects of elevation on soil microorganisms

Soil microorganisms were key drivers of biogeochemical cycles and ecosystem functions (Geisen et al., 2018). The composition and function of soil microbial communities were influenced by soil phosphorus content across most of the sampled forests in southern China (Liu et al., 2012). In this study, the 1,850 m elevation had the lowest fungal and bacterial diversity, with a significantly lower Chao1, Ace, and Shannon indices compared to other sampled elevations (Figures 3c, d). These patterns were closely linked to the low TP content at this elevation, as low P availability is known to restrict microbial growth and diversity in forest soils (Zhao et al., 2020; Liu et al., 2012). This view was also supported by the finding that both fungal and bacterial diversity increased with soil TP content in this study (Figure 6a).

Change in microbial diversity would alter microbial assembly process (Wahdan et al., 2023). Here, fungal community assembly was predominantly deterministic at 1,750 m but stochastic at the other elevations (Figure 3e), whereas bacterial community assembly was governed by stochastic processes across all elevations (Figure 3f). Deterministic fungal assembly at 1,750 m, the elevation where plant growth peaked, suggested intensified environmental filtering. This elevation represented the lower range of *A. yuanbaoshanensis* distribution, where warm, humid conditions and higher resource availability (e.g., P and K) may have intensified environmental filtering and biotic interactions among fungal taxa. Under such conditions, environmental gradients may have filtered out functionally redundant taxa, while resource competition may have favored certain life-history strategists (Velmala et al., 2014; Wu et al., 2026). In contrast, bacterial communities remained stochastically assembled across all elevations. This disparity may be due to the following factors: (i) higher bacterial dispersal rates and smaller body sizes, which promote homogenizing dispersal (Martiny et al., 2006); (ii) greater metabolic flexibility and faster generation times, buffering deterministic selection (Le Moigne et al., 2023); and (iii) lower competitive exclusion due to higher niche complementarity and metabolic redundancy (Li et al., 2026). It should be noted that the conclusions about microbial community assembly drawn in this study are exclusively based on NST. Given that NST does not account for phylogenetic or functional relationships, deterministic signals driven by trait-based selection may be overlooked. Further studies should use complementary approaches to examine this finding.

According to the oligotrophic-copiotrophic framework, copiotrophs thrive in nutrient-rich, high-carbon environments, whereas oligotrophs dominate under low-nutrient conditions (Fierer et al., 2012). There was no significant difference in the relative abundance of oligotrophs in the bacterial community among elevations (Figure 4c). Unlike this, the highest fungal oligotroph/copiotroph ratio at 1,850 m (Figure 4e) indicated oligotrophic enrichment (e.g., Basidiomycota), consistent with the highest Basidiomycota abundance at this elevation (Figure 4a) and the oligotrophic conditions. The prevalence of oligotrophic fungal community may be associated with the low P and K at this elevation, as oligotrophic fungi thrive under nutrient-limited conditions (Hu R. et al., 2023). Conversely, the lowest bacterial oligotroph/copiotroph ratio at 1,850 m reflected copiotroph enrichment, likely due to bacterial adaptation to P limitation via phosphatase secretion (Cui et al., 2022). From 1,850 to 2,050 m, the fungal ratio declined, whereas the bacterial ratio rebounded. At higher elevations, lower temperatures slowed decomposition, increasing the C/N ratio and reducing oligotrophic fungi (Hobbie et al., 2000). Meanwhile, the recovery of TP alleviated P limitation, reducing copiotrophic dominance and allowing oligotrophic bacteria to increase (Müller et al., 2017; Wang et al., 2025). It is important to note that the oligotroph-to-copiotroph ratio used in this study represents only a community-level proxy rather than a direct functional measurement at the species level. While classifications of microbial life strategies at the phylum level are inherently context-dependent and lack universal generalizability, this framework aligns with both a priori predicted ecological functions of these focal phyla and empirically observed nutrient limitation patterns in our study system. Critically, the congruence between our results and established theoretical predictions for oligotrophic environments, exemplified by Basidiomycota dominance in low-nutrient soils at an elevation of 1,850 m provides indirect but robust support for the validity and applicability of this phylum-level classification framework to our system.

Fungal-bacterial co-occurrence network analysis revealed that networks at higher elevations were smaller in scale, characterized by fewer nodes and edges, as well as a significantly higher proportion of negative links (Figure 5). This pattern suggested that while high-elevation environments constrain network size, they also intensify interactions within the network. In other words, the networks became smaller in size but more competitively intense in structure. The proportion of negative connections was often interpreted as a proxy for competitive interactions or niche partitioning (Sun et al., 2026). Under increasing environmental stress, a higher proportion of negative connections may indicate intensified competitive exclusion, but this pattern can also arise from non-competitive processes such as environmental filtering and niche differentiation among taxa (Mandakovic et al., 2023; Liu X. T. et al., 2025). At 1,750 m, the lowest proportion of negative connections indicated relatively simple microbial co-occurrence patterns, consistent with clear niche differentiation and weak competitive pressure among taxa. As elevation increased to 1,850 m and beyond, intensified P limitation and greater environmental stress likely promoted more complex interaction networks, helping microbial taxa cope with nutrient limitation (Mandakovic et al., 2023). Although studies on elevational patterns of fungal-bacterial co-occurrence networks remain limited, this research provides new insights into how cross-kingdom interactions respond to elevational gradients.

### 4.4 Relationships among plant growth, soil characteristics, and microbial community

Soil was essential for plant growth, providing essential nutrients and playing a critical role in plant development (Gao et al., 2023). In this study, both tree height and DBH increased with increasing TK content (Figure 6a). K was involved in osmotic regulation, enzyme activation, and photosynthate transport (Wan et al., 2025). Adequate K maintains cell turgor and promotes cell elongation, thereby increasing height, and enhances phloem sugar transport to support DBH growth (Pettigrew, 2008; Oosterhuis et al., 2014). Tree height and DBH also increased with the soil C/N ratio, suggesting N-limited conditions under which higher N use efficiency enhances radial growth. Conversely, an increased C/P ratio reduced both traits, indicating P limitation, as a higher C/P ratio reflects lower P use efficiency, impairing rRNA synthesis, protein translation, and cell division, thereby inhibiting cambial growth (Güsewell, 2004; Jing et al., 2017). The SEM further revealed that TP content can regulate plant growth either directly or indirectly by altering microbial community structure (Figure 7a). These results indicated that soil K and P availability were key constraints on *A. yuanbaoshanensis* growth, highlighting the importance of K and P management in forest cultivation.

Tree height increased with soil dehydrogenase activity (Figure 6a), which reflects the overall microbial metabolic activity. Higher dehydrogenase activity enhances organic matter decomposition and nutrient mineralization, promoting N and P release, improving rhizosphere nutrient supply, and supporting plant vertical growth (Wolinska and Stepniewska, 2012). Additionally, active microbial metabolism may produce auxins that stimulate cell elongation (Batista et al., 2021). Thus, this positive correlation suggestsed that soil microbial activity promotes the growth of *A. yuanbaoshanensis*.

This study found that both tree height and DBH increased with bacterial diversity but showed no significant correlation with fungal diversity (Figure 6a). High bacterial diversity likely promotes plant growth through functional groups such as N-fixing and P-solubilizing bacteria, which enhance nutrient uptake, produce phytohormones, and improve rhizosphere stability and plant disease resistance (Berendsen et al., 2012; Wagg et al., 2014). A speculative explanation for the lack of a significant effect of fungal diversity on plant growth was that opposing influences of beneficial (e.g., mycorrhizal) and pathogenic fungi may have counterbalanced each other, or that plant growth was governed by specific functional groups rather than overall diversity. This assumption was supported by the fact that tree height and DBH decreased with the fungal oligotroph/copiotroph ratio (Figure 6a), suggesting that the dominance of oligotrophic fungi in a P-poor environment was unfavorable for plant growth. Since in nutrient-limited soils, these microbes may face metabolic constraints, limiting their ability to support dominant plant species through nutrient cycling or mutualistic functions (Zhou et al., 2026). Morrissey et al. (2019) also supported that their increased relative abundance likely suppresses copiotrophic and mycorrhizal fungi, thereby reducing nutrient availability for plant growth. In contrast, copiotrophic fungi (e.g., Ascomycota) were conducive to promoting plant growth by rapidly decomposing labile organic matter and releasing nutrients in resource-rich soils (Fierer et al., 2007; Phillips et al., 2013). Correspondingly, this study found that both tree height and DBH increased with the relative abundance of Ascomycota, but declined as the relative abundance of Basidiomycota rose (Figure 6b). These results partially support hypothesis 3.

Previous studies have shown that co-occurrence networks as a key component of microbial biodiversity linking microbial communities to plant growth (Jiang et al., 2024, 2025). This study observed that tree height decreased with increasing complexity of fungal–bacterial co-occurrence networks (Figure 6a). Under environmental stress, microbes tend to form more complex and modular networks to enhance resistance and stability (Faust and Raes, 2012). However, this increased complexity may reduce nutrient mineralization and plant-growth-promoting functions due to intensified competition for resources and energy among microbial taxa (Morriën et al., 2017). Greater network complexity may also reflect a higher proportion of negative interactions (e.g., parasitism or amensalism), which can suppress beneficial microbes such as growth-promoting bacteria (Berry and Widder, 2014). In the high-elevation habitat of *A. yuanbaoshanensis*, stressors including low temperature and low pH likely drive both network complexity and growth limitation. While network complexity serves as a sensitive indicator of soil environmental stress, excessive complexity may ultimately limit plant growth by restricting nutrient supply and disrupting beneficial microbial functions. Overall, this study emphasized that high-elevation environments reduce overall network size but intensify internal competition (negative associations), and this “competitive network” ultimately suppresses plant growth.

Our SEM results also revealed contrasting direct effects of TP: positive on bacterial diversity and plant growth, yet negative on microbial community composition and fungal life-history strategy (Figure 7a). This pattern likely reflects the dual role of P in our study system. On one hand, as a limiting nutrient in alpine ecosystems (Zhu et al., 2026), higher TP availability alleviated nutrient constraints on plants and bacteria, promoting plant growth and sustaining bacterial diversity—a finding consistent with the known role of P in energy transfer and nucleic acid synthesis (Odoom and Ofosu, 2024). On the other hand, the negative TP effect on fungal community composition and life-history strategies may have operated through environmental filtering, whereby higher P levels favored copiotrophic taxa over oligotrophic specialists (e.g., Basidiomycota) better adapted to low-nutrient conditions (Carrasco et al., 2012; Yang et al., 2025). This negative TP coefficient thus reflects community restructuring via altered competitive hierarchies among fungal functional groups (Yang et al., 2025), rather than P being intrinsically harmful to fungi. Collectively, these opposing yet complementary pathways illustrate how a single factor (TP) can simultaneously benefit plants and bacteria while reshaping fungal communities, ultimately contributing to plant performance through multiple indirect routes. Several key variables beyond TP (e.g., available P, available K, root traits, mycorrhizal colonization, low-temperature stress, and wind disturbance) may also influence plant growth. These variables were not measured in the present study and therefore could not be incorporated into our SEM framework. Future studies incorporating a broader suite of environmental and biotic covariates are needed to validate and extend our findings.

### 4.5 Implications and limitations

This study revealed that elevation altered soil water content, thereby shifting TP and TK levels, which in turn regulated microbial diversity, the abundance of oligotrophic fungi, and microbial network complexity, ultimately leading to changes in plant height and DBH (Figure 7b). Despite offering novel insights into elevation regulation of the growth of endangered plants, this study has limitations that should be acknowledged. First, the co-occurrence results should be viewed as preliminary descriptive patterns rather than robust quantitative descriptions of community structure, as the networks constructed here are mainly based on statistical analysis and do not directly demonstrate real biological interactions among microbial taxa. Second, the SEM was constructed with a limited effective sample size (*n* = 12), which may limit both the statistical power of the analyses and the generalizability of the path coefficients. Third, in addition to moisture, temperature is another critical driver of soil microbial activity and plant growth. However, we note that temperature was not directly measured during sampling. Given that low temperatures at high elevations can directly constrain enzymatic activity, plant metabolism, and microbial function, temperature treatments should be designated as a key priority in future controlled experiments. Fourth, samples were collected only in December 2022, providing a winter snapshot that cannot capture seasonal dynamics, and replication was also limited, constraining statistical power and generalizability. Fifth, P limitation was inferred indirectly from TP and stoichiometric ratios, so our inference of P limitation requires direct empirical validation in future studies. Sixth, while this study reveals clear elevational patterns in the growth of *A. yuanbaoshanensis* and its associated influencing factors, the current explanations remain largely speculative without quantitative partitioning of factor contributions. Future studies incorporating manipulative experiments (e.g., transplantation and warming) are critically needed to test these factors and to partition the relative contributions of abiotic vs. biotic drivers along elevational gradients. *Ex situ* conservation efforts at different elevations require a clear understanding of humidity, soil nutrient conditions, and microbial community structure. Therefore, quantitative controlled experiments are needed to gain a deeper understanding of how environmental factors affect the growth of this endangered plant.

## 5 Conclusion

Elevation significantly influences the growth of the endangered *Abies yuanbaoshanensis*, as well as soil physicochemical properties and microbial communities. This study suggests that 1,750 m was the optimal elevation for *A. yuanbaoshanensis* growth, whereas growth at 1,850 m tends to be lower, which may be partly explained by elevated soil water content and reduced availability of TP and TK availability. The study further elucidated the mechanisms underlying the species' endangerment: low phosphorus availability indirectly limits plant growth by selectively promoting the dominance of oligotrophic fungi and intensifying competitive interactions within the microbial network, thereby constraining plant growth. This causal chain highlights that water-mediated phosphorus limitation not only directly impairs plant nutrition but also indirectly affects growth by restructuring soil fungal communities. These findings suggest that altitudinal zones and associated soil conditions must be prioritized in conservation efforts for *A. yuanbaoshanensis*—suitable habitats are critical for the expansion of wild populations, while assessments of soil nutrients and microbial communities are essential for *ex situ* conservation. Future controlled experiments are needed to clarify how environmental factors regulate the growth of *A. yuanbaoshanensis* to support population restoration.

## Data Availability

The datasets presented in this study can be found in online repositories. The names of the repository/repositories and accession number(s) can be found in the article/supplementary material.
